# A Framework to Identify Antigen-Expanded T Cell Receptor Clusters Within Complex Repertoires

**DOI:** 10.3389/fimmu.2021.735584

**Published:** 2021-11-30

**Authors:** Valentina Ceglia, Erin J. Kelley, Annalee S. Boyle, Sandra Zurawski, Heather L. Mead, Caroline E. Harms, Jean-Philippe Blanck, Anne-Laure Flamar, Jung Hwa Kirschman, Paul Ogongo, Joel D. Ernst, Yves Levy, Gerard Zurawski, John A. Altin

**Affiliations:** ^1^ Baylor Institute for Immunology Research, Dallas, TX, United States; ^2^ Université Paris-Est Créteil, Sciences de la Vie et de la Santé, Créteil, France; ^3^ Vaccine Research Institute, INSERM, Unité U955, Institut Mondor de Recherche Biomédicale, Créteil, France; ^4^ Translational Genomics Research Institute, Flagstaff, AZ, United States; ^5^ Department of Medicine, Division of Experimental Medicine, University of California, San Francisco, San Francisco, CA, United States

**Keywords:** T cell receptor (TCR), T cell responses, vaccines, dendritic cells, monoclonal antibodies, TCR sequencing

## Abstract

Common approaches for monitoring T cell responses are limited in their multiplexity and sensitivity. In contrast, deep sequencing of the T Cell Receptor (TCR) repertoire provides a global view that is limited only in terms of theoretical sensitivity due to the depth of available sampling; however, the assignment of antigen specificities within TCR repertoires has become a bottleneck. This study combines antigen-driven expansion, deep TCR sequencing, and a novel analysis framework to show that homologous ‘Clusters of Expanded TCRs (CETs)’ can be confidently identified without cell isolation, and assigned to antigen against a background of non-specific clones. We show that clonotypes within each CET respond to the same epitope, and that protein antigens stimulate multiple CETs reactive to constituent peptides. Finally, we demonstrate the personalized assignment of antigen-specificity to rare clones within fully-diverse uncultured repertoires. The method presented here may be used to monitor T cell responses to vaccination and immunotherapy with high fidelity.

## Introduction

The identification within complex repertoires of T cells for a specific target of interest is an essential immunological capability used to diagnose infection (1) and measure the immunogenicity of vaccines and immunotherapies (2). Current methods for quantifying rare antigen-specific T cells include assays that measure antigen-stimulated cytokine production (e.g., immunospot assays and flow cytometric detection of intracellular cytokines (3, 4)), as well as assays that use labeled peptide:MHC probes to directly detect antigen-binding T cells (5). Although widely useful, the ability to multiplex these assays across targets is limited, as is their sensitivity to detect rare T cell responses.

T cells recognize MHC-restricted peptide antigens by means of the heterodimeric T Cell Receptor (TCR), encoded by somatically-diversified α and β loci (6). The rearranged TCR α:β sequence pair completely determines a T cell’s specificity, and current technologies enable >1e7 unpaired or >1e4 paired TCR chains to be routinely sequenced from a sample (7). In contrast to traditional methods of antigen-specific T cell detection, deep sequencing of TCRs can reveal complete repertoires with high sensitivity. However, the ability to confidently assign antigen reactivities to (or ‘decode’) particular TCR sequences within this repertoire has become a bottleneck.

One approach to decoding the repertoire, ‘exposure association’, involves associating the incidence of particular clonotypes (e.g., defined at the CDR3β amino acid sequence level) with antigen exposure status within a cohort of individuals. This approach has the potential to reveal ‘public’ sequences that are enriched in exposed subjects and has been used to accurately classify cytomegalovirus (CMV) serostatus (8). More recently, it was used to diagnose SARS-CoV-2 infection (9). The ability to discover antigen-associated public clonotypes has powerful diagnostic potential, however, the associations discovered have generally been too weak to allow high-confidence assignment of antigen-specificity to particular public clonotypes within any given individual. This approach is also limited by a requirement for large cohorts of exposed and unexposed individuals to identify sequences with statistical confidence.

A second approach, ‘probe association’, involves the use of probes to isolate T cells that recognize defined antigens within particular samples. Multimerized peptide:MHC probes have been used for decades to identify and isolate T cells in an antigen-resolved fashion (5), including in combination with antigen-driven expansion (10). Methods combining antigen restimulation with the detection of upregulated cellular response markers can also be used for this purpose (11, 12). Although these approaches allow a powerful interrogation of the T cell response, antigen-specific cells against non-specific background binding are rare, meaning some memory T cell responses are below the limit of detection, and the peptide:MHC multimer approach depends on the *a prior* identification of appropriate peptide:MHC combinations.

Thirdly, ‘sequence-based prediction’ describes a new family of methods in which growing catalogs of defined TCR:antigen combinations are used to train machine learning algorithms to predict specificity directly from TCR sequences (13–15). These have great potential to enable generalizable decoding of the repertoire, especially as the training datasets grow, however they do not yet enable the confident assignment of specificities within deep repertoires using TCR sequences alone.

The present study developed an alternative approach to decoding TCR repertoires. In this method, rare T cells are clonally expanded by antigens of interest in culture, subjected to bulk TCR sequencing, and clonal frequencies were analyzed using a similarity-based clustering approach to identify and organize families of antigen-responsive clonotypes against the majority of irrelevant sequences.

## Materials and Methods

### Anti-hCD40 Monoclonal Antibodies

The generation and screening strategies for making in-house recombinant anti-human CD40 12E12, 11B6, and 11B6-CD40L human IgG4 antibodies fused to dockerin at the H chain C-termini are described in previous studies (16–18). The methods for expression vector and protein production *via* transient or stable CHO-S (Chinese Hamster Ovary cells) transfection and quality assurance, including CD40 binding specificity, are described in other studies (16, 17, 19). Cohesin-Influenza Matrix 1 (Flu M1) protein is described in (16).

### Donors

Cryopreserved human PBMC from normal donors were sourced commercially (AllCells, CA. ND1001 ID:A5983, ND1002 ID:9441, ND1004 ID:10504, ND1005 ID:10002, ND1007 ID:11588). Donor #30115 was a subject enrolled in a cohort study in Kenya, determined to have latent Mtb infection based on a history of tuberculosis exposure, a positive Quantiferon-TB Gold Plus assay, and the absence of any clinical symptoms of active tuberculosis. The sample was provided de-identified without any Protected Health Information, and the research was therefore considered not to be human subjects research. PBMCs from HIV-1 infected donor A12 under combined anti-retroviral therapy (cART) were prepared as described in (17) from apheresis collection as approved by the Baylor Research Institute Institutional Review Board. The HLA typing of all donors is provided in Supplemental Table 1.

### CD40-Targeting Technology

The present work utilized anti-CD40 antibody-directed targeting of antigens to facilitate the expansion of antigen-specific T cells within PBMC cultures, although we also provide examples with cultures driven by antigen-derived peptides or with bacterial culture lysate. CD40-targeting technology has been well described previously, and this work uses an anti-human CD40 IgG4 antibody format either directly fused to antigen *via* H and or L chain constant region C-termini (20) or anti-human CD40 hIgG4 fused to a dockerin domain (Doc), permitting non-covalent attachment of independently produced cohesin-antigen fusion protein (16). The anti-human CD40 11B6-CD40L hIgG4.Doc reagent used in the Flu M1 experiments is a highly activating CD40-targeting reagent that can augment at very low doses antigen-specific CD8^+^ T cell responses *in vitro* while maintaining CD4^+^ T cell responses (18). Differences in the properties of CD40 targeting *via* the anti-CD40 12E12, 11B6, and 11B6-CD40L antibodies are detailed extensively in Ceglia et al. (18). Production of anti-CD40 targeting antibody fusion proteins is done *via* transient transfection (e.g., *Trans*IT-PRO^®^ Transfection Kit, Mirus) into mammalian CHO cells followed by Protein A affinity purification using expression vectors and antibody sequences described in previous studies (18, 20). H6 or EPEA-tagged Cohesin-antigen fusion proteins can be produced in either mammalian or in *E. coli* expression systems and purified, respectively, by metal or C-tag (Thermo-Fisher) affinity as described (16).

### T Cell Expansion Assay

PBMCs were thawed with 50 U/ml benzonase^®^ nuclease (Millipore, cat 70746), washed and rested overnight in RPMI 1640 enriched with 100X PenStrep (Gibco, 15140-122), 100X Hepes Buffer (Gibco, 15630-080), 100X Non-essential amino acids (NEAA) (Gibco, 11140-050), 100X Sodium Pyruvate (Gibco, 11360-070), 1000X 2-Mercaptoethanol (Gibco, 21985- 023), 100X Glutamax (Gibco, 35050-61) (herein called complete RPMI 1640) with 10% AB serum (GemCell, 100-512) in a 37°C 5% CO_2_ incubator. The following morning, the cells were cultured at a concentration of 2e6 cells/mL at 37°C in 1 mL complete RPMI 1640 + 10% AB serum in a 24 well flat bottom plate. Cells were treated with anti-CD40 non-covalently linked to a Cohesin Influenza Matrix1 (Coh-Flu M1) protein (16), Coh-Flu M1 alone, or with 1 μM of selected Flu M1 peptides (BEI Resources, Cat NR-21541) (peptide sequences provided in Supplemental Table 2), Mtb whole cell lysate (BEI resources: NR-14822), or 10 nM anti-CD40.HIV5pep (17) depending on the experiment. After forty-eight hours, 1 mL of complete RPMI 1640 with 10% AB serum and IL-2 (Proleukin, Sanofi) at a final concentration of 100 U/mL was added to each well. Half the media was changed on day 4 and day 6, adding fresh IL-2. On day 10, cells were harvested and washed twice in PBS with 2 mM EDTA. For RNA sequencing analyses, cells were spun down and the supernatant was removed to either store the cells at -80°C before proceeding with the analyses either as a pellet or resuspended in RLT (Qiagen, cat 79216) + 1% 2-mercaptoethanol. For intracellular staining (ICS) or Luminex™ analyses, cells were instead resuspended in complete RPMI 1640 + 10% AB serum in 50 mL tubes, counted, and rested overnight at 37°C. The following day, cells were plated in a 96 well plate V bottom in 200 μL volume per well and re-stimulated with 2 μM Flu M1 peptides or controls for one hour in the case of ICS readout and up to 48 hours for Luminex™ analyses, at 37°C. Peptides were used in clusters named C1, C2, and C3 composed by, respectively, peptides 1-20, 21-40, and 41 to 60, or as single peptides or as small clusters of two or three overlapping peptides, used depending on the experiment. In the case of ICS, after one hour 0.175 μL of Golgi Stop (BD Golgi Stop, Cat 51-2092KZ) and 0.45 μL of Brefeldin A (BFA) (BD Cat 420601) were added and the cells were incubated for an additional 4 hours. Subsequently, cells were spun down and surface and intracellular staining were performed as described below gating on singlets, live cells, CD3^+^ followed by identification of TNFα^+^ and INFγ^+^ in both CD4^+^/CD8^-^ and CD4^-^/CD8^+^ cells. Cells analyzed by Luminex™ were instead spun down after the re-stimulation time and the supernatant was analyzed for secreted cytokines (21). For our study, we screened a number of normal HLA-A*02^+^ PBMC donors for a diversity of Flu M1 responses (Supplemental Table 3) and selected ND1004 and ND1005 which, respectively, had dominant CD4^+^ or CD8^+^ Flu M1-specific T cell responses, as the primary focus of our TCR analysis. When appropriate, data are presented as means (± SEM). Statistical significance was determined by Student’s t test. A P value of < 0.05 was considered statistically significant. GraphPad Prism^®^ software was used for statistical calculations.

### Surface and Intracellular Staining

Human cells were first stained for surface markers. Human cells were transferred to a V bottom plate, washed twice in PBS, and incubated for 20 minutes at 4°C with Live/Dead™ Fixable Aqua Dead Cell Stain Kit (Thermo Fisher Scientific, Cat. L34965) at a 1:50 dilution in a volume of 50 μL. Cells were washed twice with PBS and incubated for 30 minutes on ice with a mix of antibodies in a volume of 50 μL. After 30 minutes of incubation on ice with the antibodies for surface staining, cells were washed in PBS twice and resuspended in Cytofix/Cytoperm™ (BD Biosciences) for 20 min at 4°C, followed by three washes in 1X Permwash (BD Biosciences). Cells were subsequently incubated at room temperature covered from light in 1X BD Permwash with the antibody mix for intracellular cytokines. Following the incubation time, cells were washed three times in 1X BD Permwash and resuspended in BD stabilizing fixative (BD Biosciences) diluted 1:3. All analysis plots were pre-gated on live (using Live/Dead stain) and singlet events. Cells were analyzed with a FACSCanto II or an LSR Fortessa (BD Biosciences). Data were analyzed with FlowJo^®^ Software. The following antibodies were used: hCD3-BV711, clone UCHT1, ref 563725 (BD) or hCD3-PerCP clone SK7, ref 347344 (BD), hCD4-Pe-Cy7 clone SK3, ref 34879 (BD), hCD8-PacBlue clone 3B5, ref MHCD0828 (Invitrogen), hTNFα-APC clone RUO, ref 340534 (BD), and hINFγ-PE clone RUO, ref 340452 (BD).

### Isolation of Antigen-Binding T Cells

~ 35e6 of antigen-expanded cells were transferred to a 96-V bottom plate, washed with filtered PBS + 2% FBS solution (10e6 control non-antigen expanded cells were separately stained as below for control gating and tetramer comparison). After centrifugation 5 μL FcR block, 175 μL 2% FCS in PBS, 20 μL Flu-M1 Tetramer-PE were added with gentle mixing (volume was reduced to 50 μL for ‘cells only’ using 10 μL of Flu-MI tetramer). After 30 mins on ice in the dark, the plate was centrifuged and the pellet was washed with 200 μL PBS, centrifuged again, and for the antigen-expanded cells, the additional stain was added with 200 μL PBS with 4 μL of Aqua (reconstituted in 50 μL DMSO). For the control cells, 50 μL PBS with 1 μL of Aqua was used. After 30 mins on ice in the dark, cells were washed with 200 μL of 2% FCS in PBS, centrifuged, then stained with surface markers, in 200 μL in 4 lots of 50 μL of surface stain cocktail mix or 50 ul for the control cells. The mix was formulated in 2% FCS in PBS with 41.3 μL x4 = 165.2 μL; CD4-PerCP-Cy5.5: 0.3 μL x4 = 1.2 μL;CD19-APC: 2.5 μL x4 = 10 μL; CD14-APC:0.3 μL x4 = 1.2 μL; CD16-APC: 2.5 μL x4 = 10 μL; CD8-APC-Vio770: 0.6 μL x4 = 2.4 μL; CD3-PB: 2.5 μL x4 = 10 uL. The antibodies used or staining were: anti-human CD14 APC MHCD1405 (Clone TuK4) (0.3 μL/50 μL staining volume) Invitrogen; anti-human CD16 APC MHCD1605 (Clone 3G8) (2.5 μL/50 μL staining volume) Caltag; anti-human CD19 APC 555415 (Clone HIB19) (2.5 μL/50 μL staining volume) BD Pharmingen; anti-human CD8 APC-Vio770 170-081-073 (Clone BW135/80) (0.6 μL/50 μL staining volume) Miltenyi Biotec; anti-human CD4 PerCP-Cy 5.5 552838 (Clone L200) (0.3 μL/50 μL staining volume) BD Pharmingen; anti-human CD3 Pacific Blue 558124 (Clone SP34-2) (2.5 μL/50 μL staining volume) BD Pharmingen; Live/Dead Aqua was LIVE/DEAD Fixable Aqua Dead Cell Stain Kit: Invitrogen L34966. Tetramer was iTAg MHC Tetramer HLA-A*02:01 Influenza M1 GILGFVFTL-PE TB-0012-1 MBL. The staining was for 20 mins on ice in the dark, followed by centrifuging the plate, discarding liquid, and washing with 200 μL 2% FCS in PBS. Cells were resuspended and transferred to a FACS tube for sorting in a final volume of 500 μL. Sorted cells were directed into 500 μL of cRPMI 10% FBS. For the compensation controls: ArC™-Beads: 1 drop positive beads were used with 3 μL concentrated L/D-Aqua, 30 mins RT, wash 2 mL PBS, resuspend 200 μL PBS, add 1drop ArC negative beads; VersaCompbeads: individual tubes for unstained beads, CD4-PerCPCy5.5 (1 μL), CD14-APC (1 μL), CD8-APC-Vio770 (1 μL), CD3-PB (1 μL). Incubation was for 20 mins at RT and 2 mL PBS was used for washing. Beads were then resuspended into 200 μL PBS. Sorting was on a FACS Aria using BD FACS Diva software for gating analysis: 9.0.1. Gating was FCS/SSC; Singlets FCS; Singlets SSC; Live/Dead Aqua; Dump channel (APC) CD19, CD14, CD16; CD3+; CD8+/CD4-; CD8+/Tet+. Following the sort, the cell pellet was spun at 2,500 RPM, washed once in 2% FCS/PBS, and 650 μL RLT (RLT Lysis Buffer, Qiagen) with 1% 2ME (2-mercaptoethanol) added with vortexing for 45 sec followed by a quick spin and freezing at -80°C. If cell numbers were less than 10e6, only 350 μL of RLT with 1% 2ME was used.

### T Cell Receptor Sequencing

RNA isolation from cell pellets stored at -80° Celsius was performed using an AllPrep DNA/RNA Mini Kit (Qiagen). RNA quality was evaluated with an Agilent 2100 Bioanalyzer RNA pico kit (Agilent Technologies) prior to sequencing library preparation. T cell receptor sequencing libraries were prepared with the SMARTer Human TCR α/β Profiling Kit (catalog number 635015, Takara Bio USA, Inc.) according to manufacturer’s instructions with the exception of excluding the third and fourth bead size selection steps listed in Table 3 of the kit manual. Sequencing libraries were quantified using Kapa qPCR MasterMix (catalog number KK4973) on a QuantStudio7 Flex Real Time PCR System (Applied Biosystems by Thermo Fisher Scientific, Inc.). Libraries from different T cell cultures were pooled and 14 pM final library was added to the flow cell with 10% PhiX. Libraries were sequenced with MiSeq Reagent Kit v3 600 cycles (Illumina) to obtain 300 base-pair, paired-end reads. For the HIV-1 analysis, frozen cell samples were sent to Adaptive Biotechnologies Corporation for multiplexed PCR of genomic TCRβ. Numbers of input cells and sequencing read depths are provided in **Supplemental Table 4**.

### Analysis of TCR Sequences

The raw sequencing data for each sample were mapped to germline segments using *mixcr* (MiLaboratory, version 3.0.11), to generate a clonotype list in which each entry is characterized by a unique combination of V and J segments and the CDR3 nucleotide sequence. For each sample, shortlists were constructed from the 1000 most frequent TCRα and TCRβ clonotypes, respectively, and all pairwise distance measurements were made on each shortlist using the v1 TCRdist metric described previously (22). Hierarchical clustering was then performed on each set of distances using the *hclust* function in R, and clusters were identified at thresholds of 0, 5, 10, 15, 20, 25, 30, 35, 40, 45, and 50 using the *cutree* function. Each cluster was parameterized by its number of members, as well as the geometric mean frequency of its members. Significance was assigned to each cluster by determining the frequency with which clusters containing the same number of members, and a greater or equal mean frequency, were observed within 1000 random trials. The random trials used randomly-generated TCR clonotypes of the corresponding chain, constructed using the 212,651 α and β sequences published in Howie et al., 2015 (23) as a base. Each TCR sequence from this public dataset was decomposed into 14 elements using mixcr (*bestVHit, V3dels_or_Ps, nSeqVPSegment, nSeqVDJunction, nSeqDLeftPSegment, D5dels_or_Ps, bestDHit, D3dels_or_Ps, nSeqDRightPSegment, nSeqDJJunction, nSeqJPSegment, J5dels_or_Ps, bestJHit, nSeqVJJunction*). These were permuted to generate each new random TCR. For each trial, 1000 such TCRs were generated and each assigned a frequency from the 1000-member clonotype shortlist being tested, before being clustered as described above. Clusters were considered significant at p<0.01 (i.e., <10 occurrences in the 1000 random trials). Clonotypes from significant clusters detected across all TCRdist thresholds were combined into a single master clonotype list and reclustered at the maximum threshold of 50 for final output. R Code is available at https://github.com/TGenNorth/TCR_framework.git. Raw sequence data is available at SRA (https://trace.ncbi.nlm.nih.gov/Traces/sra/) under BioProject: PRJNA752634.

## Results

### Optimizing the Conditions for Whole Protein Antigen-Driven *In Vitro* Expansion of T Cells

The model we used for this study was the steady state memory T cell repertoire specific to Influenza matrix protein (M1) in healthy adult donors. M1 is >90% conserved across strains and dominates the cross-reactive memory CD4^+^ and CD8^+^ T cell repertoire in healthy individuals (24). Thus, repeated seasonal infections and vaccination account for the presence of M1-specific T cell memory in most healthy donors (25, 26). We and others have demonstrated that linking peptide or whole protein antigens to antibodies directed to Dendritic Cell (DC) receptors such as CD40 can efficiently potentiate antigen-presentation, resulting in efficient expansion of both CD4^+^ and CD8^+^ T cells across multiple epitopes and HLA specificities within *in vitro* culture systems (16, 17, 27). Our previous study described a convenient method for non-covalent assembly of anti-Dendritic Cell (DC) antibodies and antigens using a bacterial dockerin (doc) domain fused to the antibody heavy chain C-terminus, and antigen such as Flu M1 fused to a cohesin (coh) counter-domain (16).

**Figure 1:**
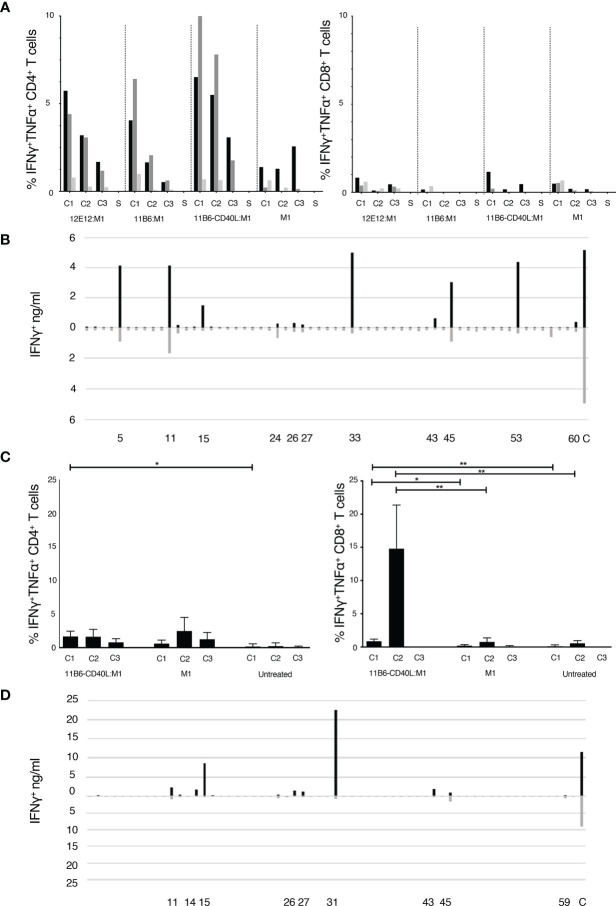
**(A, C)** Expansion of epitope-reactive T cells by M1 protein formulations. Analysis of Flu M1-specific T cell responses in a day 10 ND1004 **(A)** and ND1005 **(C)** PBMC culture stimulated with CD40-targeted Flu M1 protein. The left panel shows the % of IFNγ^+^ and/or TNFα^+^ antigen-specific CD4^+^ T cells as determined by ICS analysis. Baseline values for solvent (S, no peptide) controls were subtracted. In **(A)** cultures contained a dose range (1 nM, black bars; 0.1 nM grey bars; 0.01 nM light grey bars) of CD40-targeting antibodies conjugated to cohesin Flu M1 or Flu M1 alone (M1), while in **(C)** only one dose (1 nM) was tested. The right panel shows analogous data for % of IFNγ^+^ and/or TNFα^+^ CD8^+^ T cells. In **(A)** baseline S values for the CD4^+^ were 0.43 ± 0.14%, and for the CD8^+^ were 0.8 ± 0.4%. Compared to a starting input of PBMCs, the end stage 10 day cultures increased in total numbers as follows for, respectively, the 1, 0.1, and 0.01 nM conditions: anti-CD40 12E12:M1 5.6, 3.6,1.6-fold; anti-CD40 11B6:M1 12.5, 2.5, 0.9-fold; anti-CD40 11B6-CD40L:M1 7, 2.7 1-fold; and M1 1.5, 0.9, 0.8-fold. In **(C)** the data show results from 4 independent experiments with ND1005. Values for solvent without peptide stimulation (S) were subtracted from each peptide stimulation point; baseline S values for the CD4^+^ between 0.1 and 1, and the CD8^+^ between 0.3 and 2. Cells after 10 days expanded 6.6 ± 2.4 fold with anti-CD40 11B6-CD40L:M1 and 3.3 ± 1.2 fold with M1 alone compared to cells alone. *P ≤ 0.05, **P ≤ 0.01. The CD4^+^ T cell two-tailed T test comparison is between data for anti-CD40 11B6-CD40L:M1 and cells alone; there were no other significant differences in the CD4^+^ T cell responses. The CD8^+^ T cell two-tailed T test comparison is between data for anti-CD40 11B6-CD40L:M1 and Flu M1 and between data for anti-CD40 11B6-CD40L:M1 and cell alone; there were no significant differences in the responses to Flu M1 compared to cells alone. Statistics were calculated between cells re-stimulated with the same FluM1 cluster. **(B, D)** Flu M1 peptide-specific T cell repertoire of ND1004 (B) and ND1005 (D) expanded by anti-CD40-11B6-CD40L Flu M1 targeting.

We cultured Peripheral Blood Mononuclear Cells (PBMCs) obtained by apheresis of normal donors (ND) with dose ranges of a cohesin-Flu M1 fusion protein alone (Flu M1) or in complex with three different CD40-targeting antibody vehicles. After an expansion culture period of 10 days, cells were harvested and re-stimulated with 3 pools of overlapping 15 mer peptides covering the entire Flu M1 protein. They were then analyzed by Intracellular Cytokine Staining (ICS) for peptide-elicited production of intracellular IFNγ and TNFα. **Figure 1** shows that in ND1004, Flu M1-specific CD4^+^ T cells from epitopes within all three M1 regions were elicited with the CD40-targeted antigen being 10-100-fold more efficacious than Flu M1-stimulation alone. Up to 20% of the T cells in the 10-day culture with 0.1 nM anti-CD40 11B6-CD40L:Flu M1 stimulation produced IFNγ and/or TNFα specifically in response to Flu M1 peptides *versus* <1% elicited by 0.1 nM untargeted Flu M1. This is consistent with other data showing the high *in vitro* efficiency of targeting antigens to CD40 in PBMC cultures (17, 28). In contrast, in this donor Flu M1-specific CD8^+^ T cells were not significantly expanded in any of the conditions (**Figure 1A**) with responses below 2% and no clear trends related to stimulation condition or dose.

To ascertain the breadth of the expanded Flu M1-specific T cell responses elicited by targeting Flu M1 with anti-hCD40 11B6-CD40L, day 10 cultures were re-stimulated with individual 15 mer Flu M1 peptides and IFNγ secretion was measured 48 hours later. **Figure 1B** shows that at least 10 Flu M1 peptide specificities were elicited by anti-CD40 11B6-CD40L:Flu M1 targeting and many of these were also detected at lower response levels by non-targeted cohesin-Flu M1.

PBMCs from a second normal donor (ND1005) were cultured with 1 nM anti-CD40 11B6-CD40L:Flu M1 complex or 1 nM cohesin-Flu M1 alone, and after an expansion culture period of 10 days, cells were harvested and re-stimulated with 3 pools of overlapping 15 mer peptides covering the entire Flu M1 protein, then analyzed by ICS for peptide-elicited production of intracellular IFNγ and TNFα. **Figure 1C** shows that in this donor anti-CD40 11B6-CD40L:Flu M1 complex elicited a low level but significant ~1% M1-specific CD4^+^ T cell response from epitopes within the C1 Flu M1 region. However, in replicate experiments, 8-21% of the CD8^+^ T cells in culture with 1 nM 11B6-CD40L:Flu M1 stimulation produced IFNγ and/or TNFα specifically in response to Flu M1 C2 peptides *versus* <1.5% elicited by 1 nM untargeted cohesin-Flu M1. The breadth of the expanded Flu M1-specific T cell responses elicited by anti-CD40 11B6-CD40L:Flu M1 and Flu M1 alone were determined in day 10 cultures re-stimulated with individual 15 mer Flu M1 peptides and then assayed for IFNγ secretion after 48 hours. **Figure 1D** shows that at least 8 Flu M1 peptide specificities were elicited by anti-CD40 11B6-CD40L:Flu M1 targeting and most of these were also detected at generally lower response levels by untargeted cohesin-Flu M1.

### Identification of Antigen-Expanded Clonotypes Within the Repertoire

The above experiments established that the two selected donors contain a broad repertoire of memory Flu M1-specific CD4^+^ T cells (ND1004) and CD8^+^ T cells (ND1005) that could be efficiently expanded *in vitro* from 10 day PBMC cultures stimulated with low doses of Flu M1 targeted to CD40 on APCs, especially *via* the anti-CD40 11B6-CD40L antibody vehicle. We also cultured cells from additional normal donors, ND1001, ND1002 and ND1007, and profiled their cytokine production (summarized in **Supplemental Table 3**) but, except for ND1007, did not pursue TCR analysis of these cultures.

To profile the TCR repertoire, we extracted RNA from the cultured cells of ND1004 and ND1005, generated a library of full-length RNA products, performed nested PCR enrichment by priming against the *TRA* and *TRB* constant regions, and sequenced the resulting amplicons. Across the 5 conditions (no-antigen, Cohesin-Flu M1 protein at 0.1 nM or 1 nM, or anti-CD40 11B6-CD40L:Flu M1 at 0.1 nM or 1 nM), we recovered a total of 187,250 and 50,762 TCRα, and 124,120 and 43,607 TCRβ productively-rearranged clonotypes (each defined as a unique combination of the CDR3 nucleotide sequence and mapped V+J segments) from ND1004 and ND1005, respectively. Strikingly, 86-94% of all detected TCRα and TCRβ clonotypes were unique to a particular culture. Moreover, even when comparing the no-antigen condition (hereafter ‘Ag–’) against the most stimulatory condition (anti-CD40 11B6-CD40L:Flu M1 at 1 nM, hereafter ‘Ag+’), there was no consistent evidence of a strong antigen-driven effect on the overall clonal frequency distributions. For example, of all clonotypes detected in either the Ag– or Ag+ condition for ND1004, 55% v 40% of TCRαs and 59% v 38% of TCRβs were uniquely observed in the Ag– v Ag+ conditions (**Figure 3A**). For ND1005, these numbers were 10% v 86% of TCRαs and 19% v 75% of TCRβs for the Ag– v Ag+ conditions, respectively. Together, these observations indicate strong culture-specific effects on clonal frequencies that are independent of the added antigen, precluding the confident assignment of antigen-specificity to clonotypes based on an analysis of frequencies alone.

**Figure 2:**
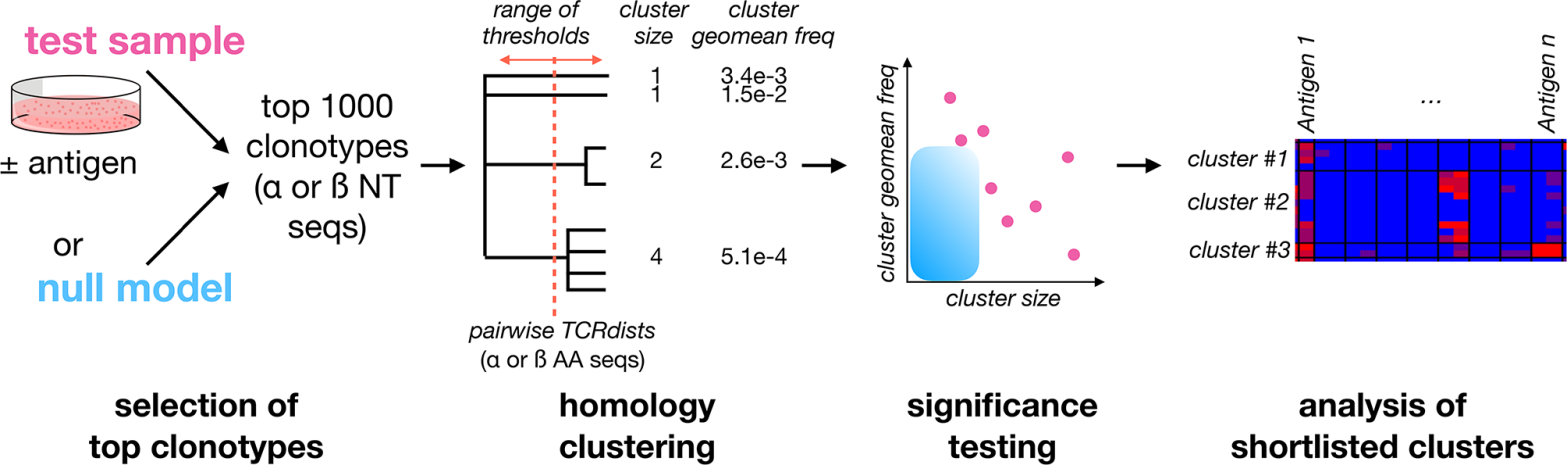
A framework for identifying Clusters of Expanded TCRs (CETs) within complex repertoires. To identify antigen-responsive clonotypes admixed within a large population of irrelevant clonotypes, the 1000 most abundant TCRα or TCRβ clonotypes for the sample of interest (resolved at the nucleotide level and quantified by deep sequencing) are analyzed by similarity-based clustering of their CDR amino acid sequences. Comprehensive pairwise similarity measurements using the TCRdist metric are used to identify clonotype clusters across a range of thresholds. The significance of each Cluster of Expanded TCRs (CET) is then quantified as the probability of observing a cluster with the same number of members, at or above its observed mean frequency, within trials of 1000 randomly-selected clonotypes. Finally, shortlisted clonotypes are analyzed for their abundance across multiple conditions.

To increase the power to identify antigen-expanded clonotypes, we reasoned that stimulation with antigen should expand families of clones that use homologous TCRs to recognize the same peptide:MHCs (22, 29). However, unlike T cells purified according to reactivity to individual antigens, we expect clonal families in expanded cultures to be admixed within a majority of irrelevant clones. To identify such families, we developed a method (**Figure 2**) based on clustering of the 1000 most frequent TCR sequences (αs and βs separately) in a sample using comprehensive pairwise homology measurements. We focused our analysis on the top 1000 clonotypes to reduce the potential for different sequencing depths to confound comparisons between samples, and to limit the computational cost of the calculations.

For each sample, we implemented the TCRdist metric (22), which provided a quantitative measure of amino acid similarity between the exposed CDR loops of any 2 TCRs. TCRdist focuses on the 4 loops of the TCR sequence that have the highest-probably of contacting the peptide:MHC: the CDR1, CDR2, CDR3 regions, as well as an additional “CDR2.5” region between CDR1 and CDR2. Each region can be identified entirely from the sequence information, using IMGT alignments. For each loop, an amino acid alignment is performed between the 2 TCRs of interest, and the values are summed to generate an overall distance score between them, ranging from 0 for TCRs with identical CDR sequences, to >100 for distantly-related TCRs.

We applied TCRdist across all possible pairs among the 1000 TCRα or TCRβ clonotypes, resulting in ~1e6 total comparisons per sample. Clusters of expanded TCRs (‘CETs’) were then identified at a range of similarity thresholds, and each CET was parametrized according to (i) its number of members and (ii) the geometric mean frequency within each overall TCR population. To exclude CETs that could occur by chance, we next estimated the significance of each CET by determining how commonly a cluster with the same number of members, and equal or greater mean frequency, arises at the same threshold based on a set of 1000 randomly-generated TCR sequences drawn from a matched underlying frequency distribution.

The sliding threshold approach is designed to enable sensitivity to TCR groups across the size:frequency spectrum: ranging from high-frequency TCR groups with few members to lower frequency groups with more members. While insensitive to antigen-specific clonotypes that do not form homology clusters, this method uses the statistical power of convergent antigen recognition to allow antigen-expanded TCRs to be confidently identified within individual samples (without an intrinsic dependency on controls or replicates), enabling condition-specific hypotheses to be tested subsequently with greater power on a more focused set of clonotypes.

**Figure 3:**
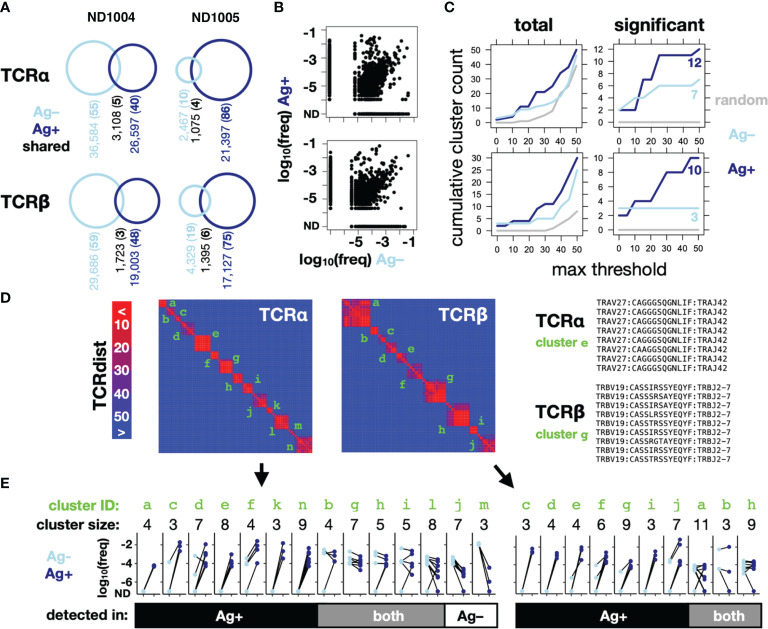
CET analysis enables the identification of Influenza M1-responsive clonotypes within complex repertoires. PBMCs from 2 normal donors were cultured with IL-2 for 10 days, in the presence (Ag*+*) or absence (Ag*–*) of CD40-targeted Influenza M1 protein (as described in Figure 1), after which RNA was extracted for amplification of TCRα and TCRβ chains, sequenced and analyzed using the scheme shown in Figure 2. **(A)** Counts of unique and shared TCRα (upper) and TCRβ (lower) clonotypes across the 2 donors. The remainder of this figure **(B–E)** shows data for one representative donor (ND1005). **(B)** Frequencies in the Ag– and Ag+ conditions of all detected clonotypes. **(C)** Cumulative number of CETs – either total (*left*) or significant (*right*) – detected up to the threshold shown on the x-axis. For comparison across thresholds, clonotypes within CETs detected up to each threshold were combined and re-clustered at the maximum threshold prior to enumeration. **(D)** Map showing pairwise TCRdist similarities of clonotypes within significant TCRα and TCRβ CETs from the analysis described in **(B)** Identifiers in green indicate CETs detected up to the maximum threshold of 50. V segment, CDR3, and J segment sequences are shown for detected clusters that correspond to a well-known HLA-A2-restricted M1 reactivity (*right*). **(E)** Clonotype frequencies in the Ag*+* and Ag*–* cultures for members of each of the significant TCRα and TCRβ CETs. Each data point represents a single clonotype within the focal CET, and line-segments connect the same clonotype between culture conditions.

Using TCRdist thresholds ranging from 0-50, we applied our clustering method to the TCRα and TCRβ clonotypes sequenced in the Ag– *versus* Ag+ conditions for ND1005 (**Figures 3B–E**), as well as to a randomly-generated control set of clonotypes. The total number of detected CETs was greater in the Ag+ compared to Ag– condition, and was lowest in the randomly-generated set (**Figure 3C**, left). When focusing only on CETs that passed the significance test (described above, based on cluster sizes and mean frequencies relative to a random model), the enrichment in the Ag+ condition was more marked, and as expected, none of the clusters detected in the random control reached significance for any TCRdist threshold (**Figure 3C**, right). CET analysis, therefore, revealed an asymmetry between the Ag– *versus* Ag+ conditions that are expected, yet much less obvious on a frequency-only analysis.

Since our method looks only for TCR clusters expanded within an individual sample, without regard to the presence or absence of antigen, the CETs identified in the Ag+ condition could represent clonotypes expanded either *in vivo* (against any antigen) or *in vitro* (against Flu M1). We resolved these possibilities by comparing the identities and frequencies of the significant CETs detected between the Ag+ and Ag– conditions. Combined across both the Ag+ and Ag– conditions, the analysis revealed 14 significant TCRα CETs, comprising 3-9 members (77 total clonotypes), and 10 significant TCRβ CETs, comprising 3-11 members (59 total clonotypes) (**Figures 3D, E**). Strikingly, 5/7 and 3/3, respectively, of the α and β CETs detected in the Ag– condition were also significant in the Ag+ condition, and their constituent clonotypes generally showed minimal differences in frequency between the 2 conditions, indicating their expansion independently of the Flu M1 antigen and likely *in vivo* prior to culture. In contrast, the majority of CETs (7/12 and 7/10 α and β, respectively) detected in the Ag+ condition were not detected in the Ag– condition, reflecting the fact that their constituent clonotypes were dramatically (100-10,000-fold) expanded in the Ag+ condition.

The largest TCRα CET contained 8 clonotypes, each comprising the *TRAV27*/*TRAJ42* segment pair with consensus CDR3 sequence ‘CAGxGSQGNLIF’. Similarly, the largest TCRβ cluster detected only in the Ag+ condition contained 9 clonotypes, each comprising the *TRBV19*/*TRBJ2-7* pair and with CDR3 consensus CASSxRSSYEQYF (**Figure 3D**, right). These correspond precisely with a known TCRα:β paired motif previously described for CD8^+^ T cells recognizing the immunodominant HLA-A2-restricted Flu M1 peptide GILGFVFTL (22), consistent with ND1005’s status as HLA-A2+ and validating the algorithm’s ability to robustly identify an expected antigen-specific TCR clonotype within the unpurified repertoire.

To verify that the CET analysis identifies clonotypes that expand during culture, we cultured cells from an additional normal donor (ND1007, also HLA-A2+) using the CD40-targeted Flu M1 antigen. In this culture, cells were harvested at 2-day intervals from day 0 to day 8 and evaluated using TCR sequencing and CET analysis. Analysis of day 8 samples that were cultured ± Ag identified 6 putative antigen-specific CETs (3α and 3β) (**Supplemental Figure 1**). Frequency tracking of the constituent clonotypes across the time series revealed consistent increases, in which the clonotypes of each CET were generally undetected at days 0 and 2, and then became detectable and progressively more frequent starting at days 4, 6, or 8. Consistent with the donor’s HLA-A2+ genotype, 2 CETs (1α and 1β) strongly matched the TCR motifs described above, which are known to recognize the HLA-A2:GILGFVFTL antigen.

### Extending Cluster Analysis to T Cell Responses Against Other Antigens

To test whether the CET identification method generalizes to other antigens and disease settings, we applied it to study T cell responses to 2 other pathogens: *Mycobacterium tuberculosis* (Mtb) (**Supplemental Figure 2**) and Human Immunodeficiency Virus-1 (HIV-1) (**Supplemental Figure 3**). PBMCs from a subject latently-infected with Mtb (donor #30115) were expanded in culture with IL-2 in the presence or absence of Mtb lysate, reflecting a crude but highly-diverse antigen formulation. At the end of the culture, TCRα and β libraries were prepared from mRNA and sequenced as previously described. Similar to what we observed for the targeted Flu M1-expanded cultures, the majority of clonotypes (92% of TCRαs and 93% of TCRβs) were uniquely detected in a single culture, and these showed no clear asymmetry between the presence or absence of antigen: 46% v 46% of total TCRαs and 45% v 48% of total TCRβs were detected uniquely in the Ag– v Ag+ conditions, respectively (**Supplemental Figures 2A, B**). In contrast, a clear distinction between conditions was observed for the number of CETs (**Supplemental Figure 2B**): with 2 v 7 TCRα CETs and 2 v 10 TCRβ CETs detected in the Ag– v Ag+ conditions. Two of the lysate-specific TCRβ CETs (‘h’ and ‘i’ in **Supplemental Figures 2C, D** comprising a total of 13 clonotypes) used the TRBV9 segment and a 14-amino acid CDR3 region beginning with the consensus sequence ‘CASSVAL’, closely matching a CD4^+^ T cell specificity group previously reported to recognize an HLA-DRB1*15:03-restricted peptide (MHVSFVMAYPEMLAA) derived from the Rv1195 Mtb gene product (29). Genotyping of donor #30115 showed the 11:02:01/15:03:01 genotype at HLA-DRB1 (**Supplemental Table 1**).

HIV-1-infected patients under anti-retroviral therapy develop a diverse array of memory T cells specific to HIV-1 antigens and loading dendritic cells *in vivo* with a mixture of HIV-1-peptide antigens *via* CD40-targeting is one strategy to expand them with potential therapeutic benefits (20). We have previously shown that a candidate HIV-1 vaccine based on anti-CD40 12E12 antibody fused to a string of five highly conserved CD4^+^ and CD8^+^ T cell epitope-rich regions of HIV-1 Gag, Nef, and Pol (αCD40.HIV5pep) expands multi-epitope HIV-1 antigen-specific CD4^+^ and CD8^+^ T cells producing multiple cytokines and chemokines in PBMC cultures (17). In donor A12 PBMC cultures stimulated for 10 days with 10 nmol/l αCD40.HIV5pep, and then re-challenged for 6 h with or without the 5 individual HIV-1 long peptides, vaccine-specific IFNγ^+^ CD4^+^ T cells were detected with gag253 peptide stimulation (2% of CD4^+^ T cells) and vaccine-specific IFNγ^+^ CD8^+^ T cells were detected with gag17 peptide stimulation (1% of CD8^+^ T cells) (17). We studied the TCR response in donor A12 PBMCs cultured with IL-2 in the presence or absence of αCD40.HIV5pep. In contrast to the previous analyses of TCRα and TCRβ RNA, here we sequenced only the TCRβ, using a library generated by multiplexed PCR of the target locus in genomic DNA (*Adaptive Biotechnologies*).

In agreement with previous observations for Influenza and Mtb antigen cultures, the cultures from donor A12 95% of the total detected clonotypes were unique to a condition and were approximately symmetric in their distribution: 55% being unique to the Ag– culture, and 40% being unique to the Ag+ culture (**Supplemental Figures 3A, B**). In contrast, our algorithm revealed 4 significant CETs (**Supplemental Figures 3C–E**), all of which were detected in the Ag+ culture, and 1 of which was also detected in the Ag– culture. In total, our data across the 3 systems demonstrates that the CET algorithm identifies TCRα and β clusters significantly enriched by antigen in the setting of diverse diseases, and uses 2 different, commonly-used strategies for sequencing the TCR repertoire. In several cases, the detected clusters match known antigen-specific motifs restricted by HLA proteins expressed by the donor.

### Characterization of TCR Clusters Across Distinct Antigens and Formulations

**Figure 4:**
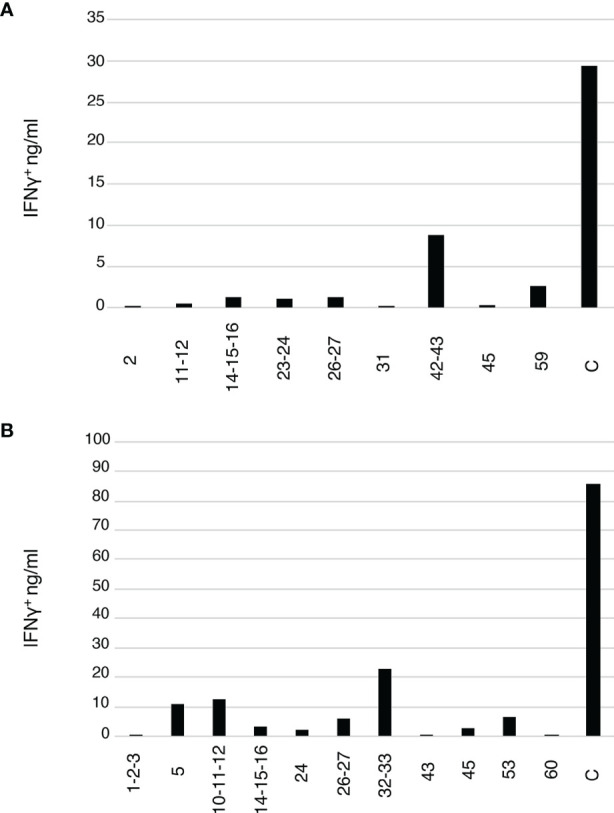
Flu M1 epitope-specific T cell repertoires expanded by single or small clusters of Flu M1 peptides. **(A)** ND1005 and **(B)** ND1004 PBMCs were cultured in complete RPMI 1640 + 10% AB serum for 10 days in the presence of IL-2, stimulated with 1 μM of either single peptides or small clusters of Flu M1 peptides. On day 11 cultures were re-stimulated with 2 μM of the matching Flu M1 peptides or PHA as a control (C) for 48 h and the collected supernatants were analyzed for IFNγ.

To test the hypothesis that the detected CETs correspond to groups of TCRs united by their antigen recognition at the epitope-level, we reasoned that the members of each CET should respond in a coordinated way when expanded with different constituent antigenic peptides from the protein antigen. We identified candidate Flu M1 antigenic peptides in our study subjects from the patterns of re-stimulated cytokine production shown in **Figures 1B, D** and used these to generate additional cultures in which T cells from the same donors were stimulated with 1 μM of either single or small clusters of overlapping Flu M1 peptides or polyclonal stimulation with phytohemagglutinin (PHA) as a positive control. After 10 days of culture, the cells were re-stimulated with the matching peptides for 48 hours or with PHA (C), and the collected supernatants were analyzed for IFNγ production (**Figure 4**). RNA from these same unrestimulated cultures, together with the original RNA samples in which T cells from the same donors were cultured with the CD40-targeted or untargeted whole Flu M1 protein, was then analyzed for the representation of TCRα and TCRβ clonotypes.

**Figure 5:**
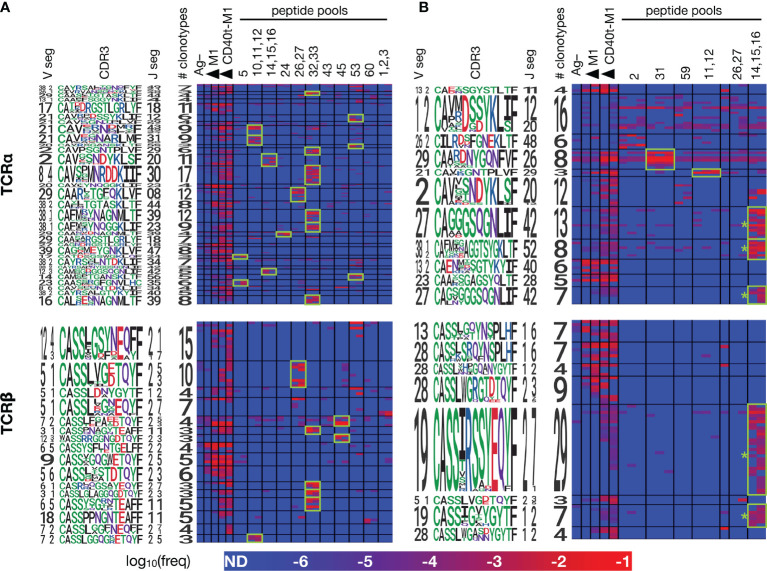
Members of each CET show coordinated responses that distinguish different forms of antigen. PBMCs from ND1004 and ND1005 **(A, B)**, respectively) were expanded in replicate cultures with influenza M1 antigen in a variety of forms – untargeted or CD40-targeted whole protein, or pools of overlapping peptides corresponding to the reactive epitopes identified in Figure 4, and then analyzed by TCR sequencing and CET identification as described previously. Significant CETs were identified in each sample individually, aggregated across all samples, and then re-clustered at the maximum TCRdist threshold of 50 for display. Each row represents a single clonotype, with CETs demarcated by horizontal black lines and labeled by logos representing their constituent V, CDR3, and J sequences. Each column represents a single culture, with conditions demarcated by vertical black lines (one replicate per column). Shown are CETs with ≥3 members and ≥100X average enrichment in any condition over the Ag– condition; highlighted in green are CET:peptide combinations with ≥100X average enrichment over the Ag– condition. * = CETs whose sequence features closely match TCRs previously described to recognize the HLA-A2-restricted GILGFVFTL antigen (22).

We applied the CET-detection algorithm described above to TCRα and TCRβ clonotypes from each condition in the 2 donors and aggregated the identified clonotype clusters across the different conditions to generate a master list for each donor that was then re-clustered for display. TCRα and β CETs whose members show an average expansion of at least 100-fold in any condition over the Ag– condition are shown in **Figure 5**. In total, we identified 11 and 8 antigen-enriched TCRα and TCRβ CETs (comprising, respectively, 88 and 70 total clonotypes) meeting those criteria in ND1004, and 31 and 16 TCRα and TCRβ CETs (comprising, respectively, 207 and 84 total clonotypes) in ND1005.

Consistent with our hypothesis, clonotypes within the identified clusters showed patterns of reactivities across the different Flu M1 antigens that were strongly-coordinated. In both donors, the anti-CD40 11B6-CD40L:Flu M1 formulation expanded the largest number of CETs, consistent with its enhanced immunogenicity compared to untargeted protein, and reflecting the cytokine production patterns that we observed (**Figure 1**). For each of the 4 donor:TCR chain combinations, the untargeted cohesin-Flu M1 protein expanded a significantly smaller group of CETs, in each case being a subset of those expanded by the targeted version of the protein. Consistent with expectations, a majority (11/17) of the peptide pools expanded at least 1 (and up to 6) discrete CETs, and there was a general correlation between the number of α v β CETs across donor:antigen combinations, the most striking example being peptide pool 32,33 which expanded 6 α and 4 β CETs in ND1004. Segment usage and CDR3 motifs were largely non-conserved across these CETs, suggesting that the peptide pool contains multiple (but nearby) epitopes, and/or that TCRs with diverse sequence features are recognizing the same peptide:MHC complex.

Conversely, cluster specificity at the peptide level was evident from the fact that a CET never responded to more than 1 distinct peptide pool. Moreover, the ‘14,15,16’ peptide pool, which covers the immunodominant HLA-A2-restricted epitope ‘GILGFVFTL’ mentioned previously, stimulated 5 CETs (3 α and 2 β – denoted by ‘*’ in **Figure 5B**) all of which correspond to previously-described TCR sequence motifs for this epitope (22). Interestingly, these CETs contained an unusually large number of clonotypes (28 TCRα and 36 TCRβ) and were most strongly expanded in the peptide-only condition, followed by the targeted-protein condition, and not significantly expanded at all in the untargeted-protein condition. This observation suggests limitations in antigen processing in the case of the whole-protein antigen, and that these might be overcome by the CD40-targeting.

To formally verify that the clonotypes revealed by CET analysis correspond to bona fide antigen-specific T cells, we performed TCR sequencing on bulk RNA extracted from HLA-A2:GILGFVFTL tetramer-binding T cells sorted from the culture of ND1005 with CD40-targeted Flu M1 antigen. The resulting clonotypes were queried against members of the 19 CETs (11 α and 8 β) identified above in **Figure 5**. This analysis revealed 45 clonotype matches, of which 44 were restricted to the 5 CETs (3 α and 2 β) identified as responsive to the ‘14,15,16’ peptide pool (**Supplemental Figure 4**). The majority of constituent clonotypes for each of these CETs was detectable in the sorted fraction.

### Quantification of Antigen-Specific Clonotypes Within Matched Uncultured Repertoires

A key motivation for developing methods for decoding TCR repertoires is to enable multiplexed and sensitive monitoring of rare T cells. Having identified sets of high-confidence Flu M1-responsive TCR clonotypes from study subjects ND1004 and ND1005, we next explored whether these responses could be detected in their original states in deep *ex vivo* samples.

Since it is theoretically possible that an unpaired TCR chain detected in an individual derives from multiple TCRs of different specificities (owing to pairing with different chain partners), we measured the frequency with which clonotypes identified by the CET analysis are observed within deep uncultured samples from both matched and unmatched donors. We reasoned that detection of these TCR clonotypes in samples from the matched, but not the unmatched, donors would indicate assay specificity, and set a limit on the frequency with which chain rearrangements could converge by chance and confound the analysis. Conversely, we surmised that the occurrence of the queried sequence in unmatched donors would indicate a specificity limit, beyond which the inferred link between unpaired clonotype sequence and antigen specificity may break down.

**Figure 6:**
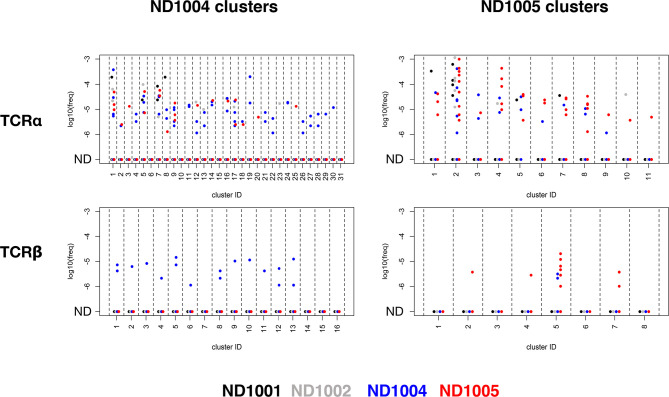
Specificity of rare antigen-specific TCRβ, but not TCRα, clonotypes within deep unenriched repertoires. TCRα and β libraries were prepared from uncultured PBMCs from 4 healthy subjects (ND1001, ND1002, ND1004, and ND1005), and deeply sequenced to generate an average of >1.3M clonotype counts per sample. Clonotypes within each of the TCRα and β CETs identified previously in ND1004 and ND1005 (Figure 5) were queried against these 4 deep, uncultured datasets by matching for nucleotide-level sequence identity. Plots show log10(frequency) of sequences in the 4 uncultured datasets (colored by donor) and organized by chain type (upper/lower), CET donor (left/right), and CET grouping (x-axis groups).

We sequenced uncultured PBMCs from a total of 4 healthy donors – the 2 characterized so far (ND1004, ND1005), and 2 additional controls (ND1001 and ND1002) – to an average depth of 1.3e6 mapped TCR clonotype reads. We then queried these 4 deep uncultured repertoires for nucleotide-level sequence matches across each of the 295 α and 154 β clonotypes contained in the Flu M1-responsive CETs that we previously identified in ND1004 and ND1005 (using the analyses described in **Figure 5**). The results are presented in **Figure 6**.

For the Flu M1-responsive TCRαs, ≥1 clonotype was detectable in ≥1 of the 4 uncultured samples in a large fraction of CETs (27/31 and 11/11 of the ND1004 and ND1005 CETs, respectively), with frequencies ranging from 1e-6 to 3e-3. While these detectable clonotypes occurred disproportionately in the samples matching the donors from which they were identified (48/83 and 35/67 for the ND1004 and ND1005 clonotypes, respectively), there was also a substantial fraction of occurrences in unmatched donors, across a similar range of frequencies.

For TCRβs, in contrast, the overall matching rates (≥1 clonotype was detectable in ≥1 donor in 12/16 and 4/8 ND1004 and ND1005 CETs, respectively) and frequencies (1e-6 to 5e-4) appeared somewhat reduced, but now these clonotypes were highly-specific for the matching donor. Among the Flu M1-responsive TCRβ ND1004 clonotypes that were also identified in an uncultured sample, all 17/17 were identified in ND1004. For ND1005, this rate of ‘matching hits’ was 10/12, with 2 ‘unmatched’ clonotypes in cluster #5 (corresponding to TCRs recognizing the HLA-A2-restricted ‘GILGFVFTL’ epitope) detected in ND1004. The antigen-specificity of these 2 clonotypes is unknown, and it remains possible that they also recognize this same immunodominant epitope. Overall, we conclude that TCRβ, but not TCRα, clonotypes assigned to antigen using the CET analysis are often detectable with high specificity in the *in vivo* state, down to frequencies ~1e-6. The observed difference in the background frequencies of the TCRα v TCRβ sequences is consistent with the greater overall diversity of TCRβ, including that contributed by the Diversity segment which is absent in TCRα.

## Discussion

The approach described here allows TCR sequences within complex repertoires to be confidently assigned to antigens of interest. Unlike existing approaches that require cell labeling and isolation, our method uses a statistical analysis of deeply-sequenced TCR repertoires in response to antigen-driven expansion. Taking advantage of the fact that individual epitope-specific immune responses often comprise groups of homologous TCRs, we integrated both TCR frequency and sequence homology information across the repertoire to identify groups of antigen-expanded clonotypes within individual samples. Using this method, we observe that the T cell response to several antigens – namely, the M1 protein from Influenza, a lysate containing Mtb proteins, and a recombinant fusion of epitope-rich regions from several HIV-1 proteins – each comprise groups of homologous clones. In a more detailed analysis of the response to Influenza M1, we showed that these clusters are raised against distinct peptide epitopes and that many of the responding TCRs are also detectable in their rare, uncultured state in *ex vivo* samples. We also showed that the number of antigen-specific clonotypes detected can be dramatically augmented by CD40-targeting.

Consistent with previous work, our analysis demonstrates that the peptide:HLA-specific T cell response within a donor frequently comprises a large number of individual clonotypes whose TCRs use convergent sequence features to recognize the antigen. The most striking example observed here is the 29-member *TRBV19+*/*TRBJ2-7+* cluster recognizing the well-documented immunodominant HLA-A*02:01-restricted GILGFVFTL peptide (**Figure 5B**). The factors contributing to the activation of such a large T cell family within an individual also likely underlie immunodominance *across* individuals – namely, a high generation probability of T cell precursors capable of recognizing the antigen, and abundant or sustained expression of the antigen during infection (4, 30). It remains to be seen how frequently and in what other settings such large intra-donor TCR clusters may arise.

The expansion-based approach described here differs in several notable ways from alternative methods for TCR mapping that use cell labeling and isolation. At a technical level, the method herein does not rely on cell isolation, nor does it require peptide:MHC multimer probes to be identified and constructed. It does, however, involve antigen expansion cultures, which may become a bottleneck when interrogating large numbers of antigens. However, like existing approaches, it is likely that the number of targets analyzed simultaneously can be increased by implementing a scheme in which antigens are multiplexed combinatorially (11). Another difference is that, unlike antigen-binding or antigen-induced marker upregulation, the use of antigen-driven *in vitro* expansion is expected to select against anergic or regulatory cells that do not divide substantially upon stimulation, and instead highlight the most proliferative elements of the response. The clonal expansion also serves as a form of signal amplification to increase the sensitivity for rare clonotypes: a prior study reported a more sensitive detection of antigen-specific clonotypes when cells were isolated according to their antigen-driven proliferation (by dilution of a CFSE marker), compared to upregulation of an activation marker or binding to a peptide:MHC probe (31).

Our CD40-targeted results indicate that stimulation with more immunogenic formulations of antigen can further increase the sensitivity with which rare clonotypes are detected. We show that anti-CD40 11B6-CD40L:Flu M1 immunogen elicits a response comprising substantially more detectable T cell clonotypes and homology clusters than the same protein in untargeted form, consistent with the observation that such targeting leads to increased T cell proliferation and cytokine production. This likely reflects a combination of CD40 activation of the APC concomitant with antigen uptake, focusing antigen to the APC *via* the anti-CD40 antibody binding, and specialized internalization into a dominantly early endosome compartment, resulting in sustained antigen presentation (18, 28). As well as increasing the power to detect antigen-responsive TCRs, this likely provides a better (e.g., as compared to stimulation with peptide pools) representation of the response that is generated *in vivo* during natural infection or vaccination.

A limitation of the method we describe here is that it is unable to assign antigen specificity to antigen-expandable T cells that do not form receptor sequence homology clusters. Studies in which individual peptide:MHC-binding T cells were isolated and sequenced have defined, for most epitopes, a core group of TCRs belonging to one or more homology groups(s), and a remainder of TCRs that do not share evident sequence similarity (22, 29). This is consistent with a model in which a given 3-dimensional peptide:HLA antigen can often be recognized by a range of TCR sequence ‘solutions’ that are non-homologous in linear sequence space, but each of which can also tolerate some degree of homologous sequence variation. The magnitude of the T cell response corresponding to any given group is likely to be a function of both (i) the degree of sequence variation that is tolerated by the structure of the antigen, and (ii) the generation/maturation probability of TCRs within the group. The same considerations suggest that the homology groups to which our method is most sensitive (namely: groups that are frequently generated and for which antigen-binding tolerates considerable sequence variation) are also the most likely groups to respond publicly across donors. Accordingly, we expect that the future application of our method to larger cohorts will reveal that many of the identifiable CETs recur across individuals with matched HLA types.

The confident assignment of single chain TCR sequences to cognate antigens is complicated by several factors, including the heterodimeric nature of the TCR, the potential of any given TCR to cross-react with diverse antigens (32), and the vast complexity of the repertoire found in any individual (33). Nonetheless, we show that unpaired α and β chains can be confidently assigned to antigen without cell isolation, and instead using statistical analysis of clonotype frequencies in expansion cultures. Moreover, our interrogation of uncultured samples indicates that nucleotide-resolved unpaired β chain clonotypes are sufficiently-specific biomarkers to enable inference of antigen-specificity within deeply personalized repertoires down to frequencies less than 1e-6. The methodology developed here may be used to derive convenient high-fidelity biomarkers of antigen-specific T cell responses in the context of infection and/or vaccination studies. For example, applying this approach to longitudinal blood draws could enable highly-sensitive, multiplexed, and antigen-resolved monitoring of the evolution of the circulating T cell response to a vaccine.

## Data Availability Statement

The datasets presented in this study can be found in online repositories. Raw sequence data is available at SRA (https://trace.ncbi.nlm.nih.gov/Traces/sra/) under BioProject: PRJNA752634. Analysis code is available at: https://github.com/TGenNorth/TCR_framework.git.

## Author Contributions

VC: Investigation, Methodology, Formal analysis, Data curation, Writing – original draft, review, and editing. EJK: Investigation, Methodology, Formal analysis, Data curation. ASB: Investigation, Methodology. SZ: Investigation, Methodology. HLM: Investigation, Methodology. CEH: Investigation, Methodology. J-PB: Investigation. A-LF: Investigation. JHK: Investigation, Methodology. PO: Provision of samples. JDE: Provision of samples. YL: Funding acquisition, Supervision, Visualization. GZ: Conceptualization, Methodology, Formal analysis, Data curation, Funding acquisition, Supervision, Writing - original draft, review, and editing. JAA: Conceptualization, Methodology, Formal analysis, Data curation, Supervision, Writing - original draft, review, and editing. All authors contributed to the article and approved the submitted version.

## Funding

We acknowledge funding support from the Roche Collaborative Research Grant to the Baylor Scott and White Research Institute (supporting the initial development of anti-CD40 antibodies with enhanced agonist potency), the Vaccine Research Institute *via* the ANR-10-LABX-77 grant, and the NIH grant U19 AI111211. The funder was not involved in the study design, collection, analysis, interpretation of data, the writing of this article or the decision to submit it for publication.

## Conflict of Interest

VC, SZ, YL, and GZ are inventors on patent application WO2020193718.

The remaining authors declare that the research was conducted in the absence of any commercial or financial relationships that could be construed as a potential conflict of interest.

## Publisher’s Note

All claims expressed in this article are solely those of the authors and do not necessarily represent those of their affiliated organizations, or those of the publisher, the editors and the reviewers. Any product that may be evaluated in this article, or claim that may be made by its manufacturer, is not guaranteed or endorsed by the publisher.
