# Supplementary material for: A Framework to Identify Antigen-Expanded T Cell Receptor Clusters Within Complex Repertoires
Source: Front Immunol. 2021 Nov 30;12:735584. doi: 10.3389/fimmu.2021.735584 (PMC8670329; doi:10.3389/fimmu.2021.735584)
Supplement: Supplementary file 1 [file DataSheet_1.pdf]

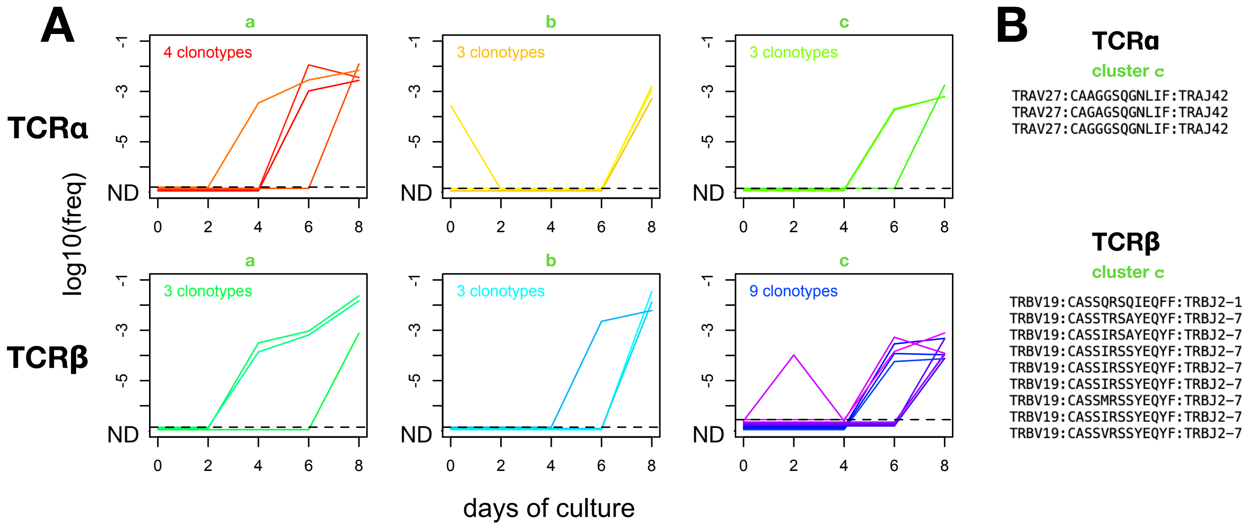

Supplemental Figure 1. Clonotype-level kinetics of CET expansion during culture with CD40-targeted M1. A Cells from donor ND1007 were expanded with CD40-targeted Flu M1 protein or no antigen as a control. At days 0, 2, 4, 6 and 8 of culture, aliquots of cells were collected and TCRs enumerated from the RNA. Analysis of the day 8 samples identified 3 TCRα and 3 TCRβ antigen-responsive CETs (1 per plot): shown are the clonotype frequencies at each timepoint of the members of these CETs (1 line per clonotype). **B** Segment usage and CDR3 amino acid sequences of clonotypes in TCRα cluster c and TCRβ cluster c, matching the motifs of TCRs previously described for $CD8^+$ T cells recognizing the immunodominant HLA-A2-restricted Flu M1 peptide GILGFVFTL.

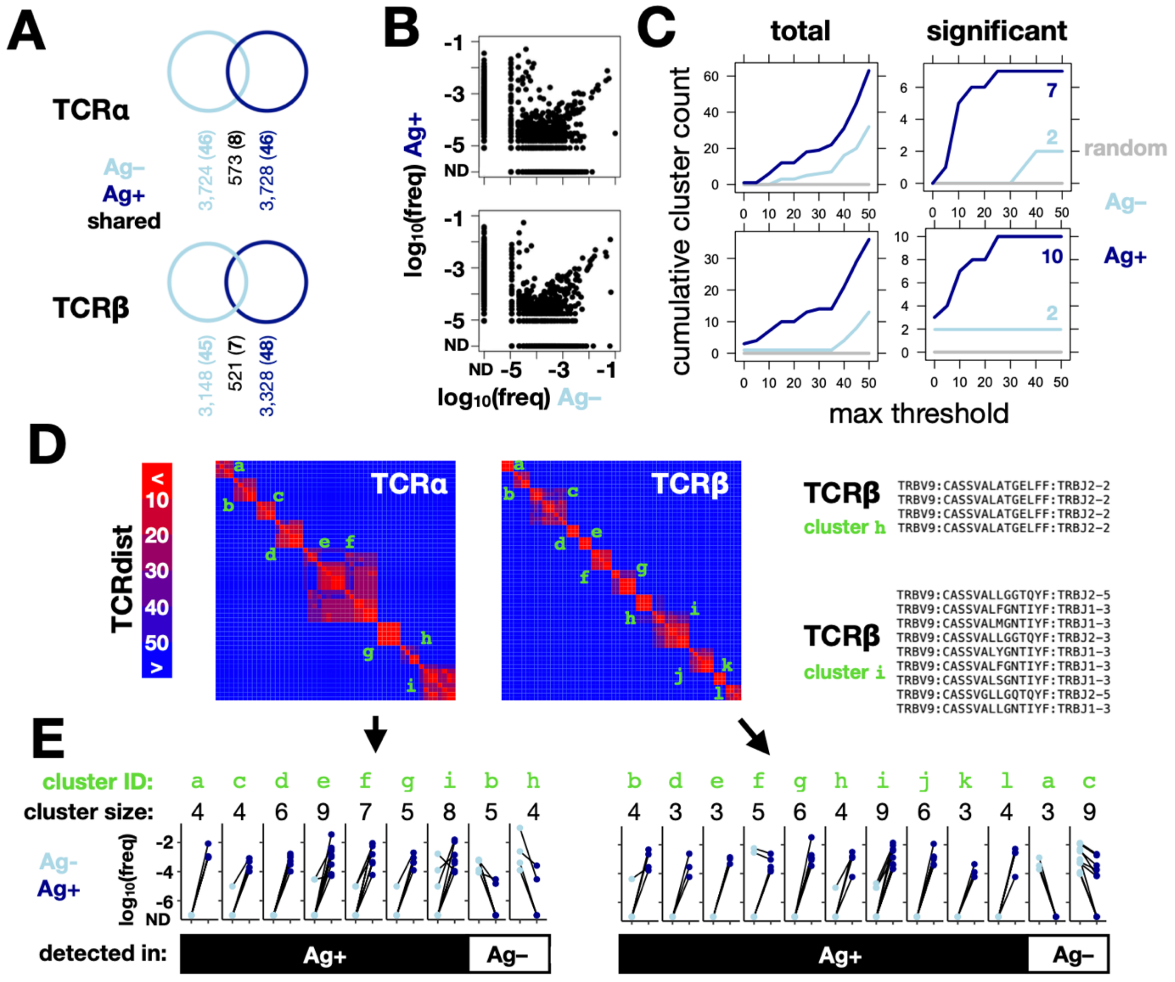

Supplemental Figure 2. CET analysis identifies Mtb-responsive clonotypes. PBMCs from a subject latently-infected with Mtb were cultured with IL-2 for 10 days, in the presence (Ag+) or absence (Ag–) of Mtb lysate, after which RNA was extracted for amplification of TCRα and TCRβ chains, sequenced and analyzed using the scheme shown in Figure 2. Panels **A-E** are exactly as described for the corresponding Influenza M1 panels of Figure 3, with the exception that the clusters highlighted at the right-hand side of **D** now correspond to a motif known to recognize the Mtb peptide MHVSFVMAYPEMLAA presented by HLA-DRB1*15:03.

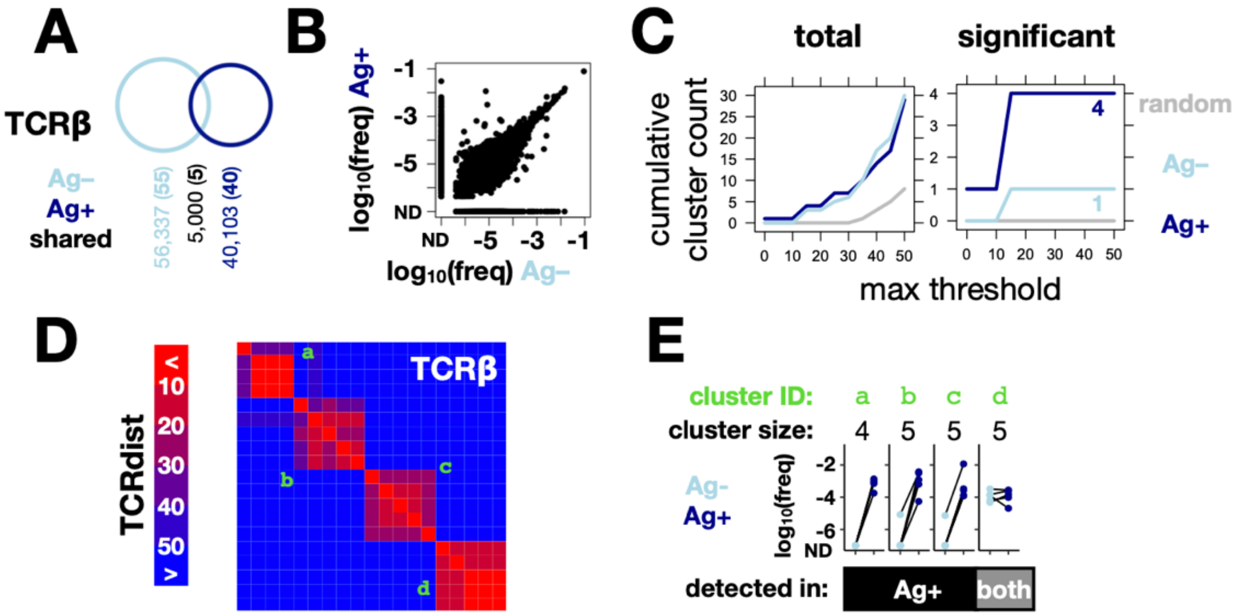

Supplemental Figure 3. CET analysis identifies HIV-responsive clonotypes. PBMCs from a subject latently-infected with Mtb were cultured with IL-2 for 10 days, in the presence (Ag+) or absence (Ag–) of a string of five conserved T-cell epitopes of HIV-1 Gag, Nef and Pol (17) after which DNA was extracted for amplification of TCRβ chains, sequenced and analyzed using the scheme shown in Figure 2. Panels **A-E** are exactly as described for the corresponding Influenza M1 panels of Figure 3.

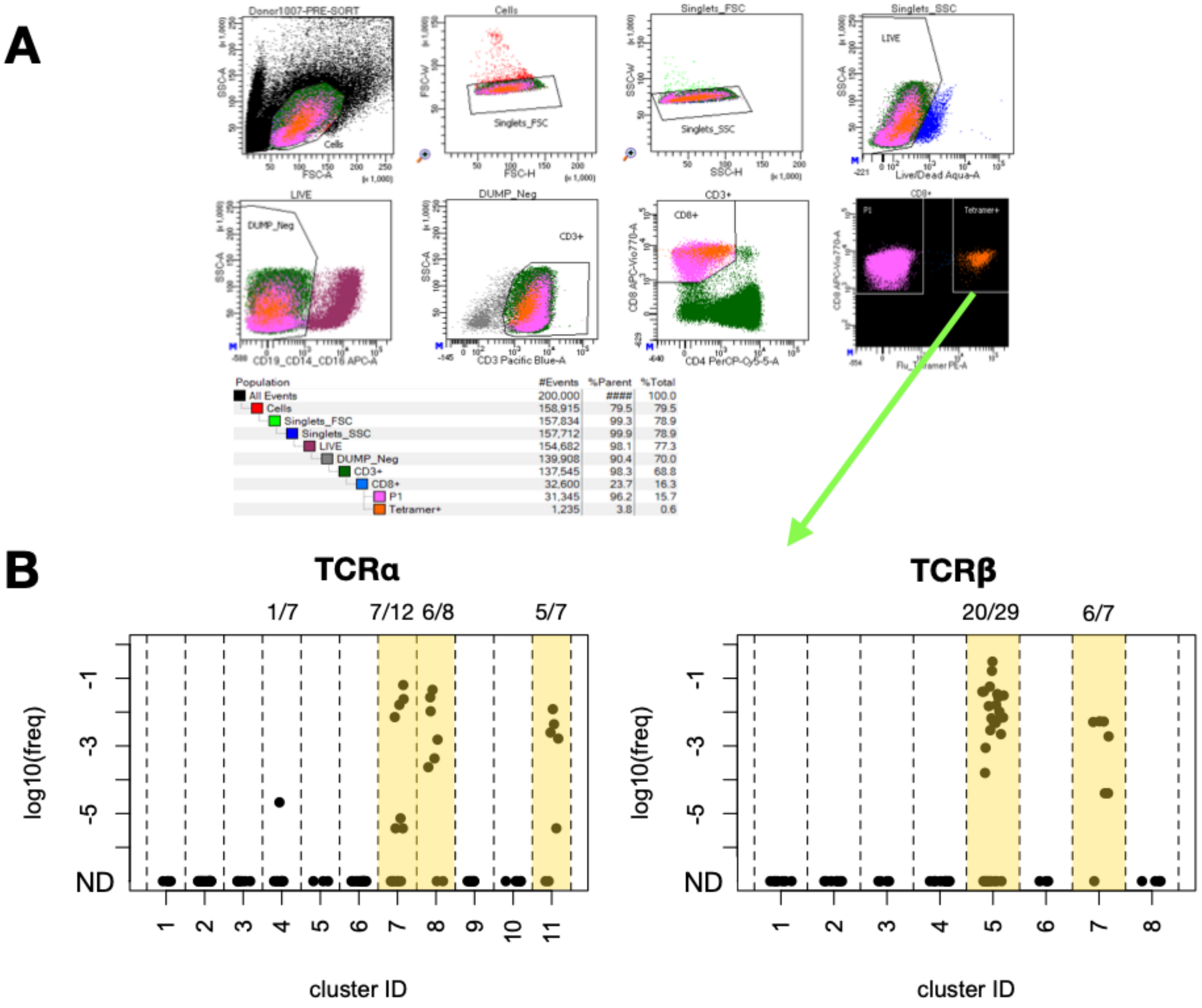

Supplemental Figure 4. Clonotypes identified by CET analysis of unsorted cultures match those identified by cell sorting based on antigen binding. A Cells from donor ND1005 were expanded with CD40-targeted Flu M1 protein. Those recognizing the HLA-A2:GILGFVFTL immunodominant M1 epitope were isolated by cell sorting using a fluorescent peptide:MHC tetramer, using the gating strategy shown. **B** TCRs from sorted cells were enumerated from the resulting total RNA as described. Shown are the frequencies among total TCRα (*left*) or TCRβ (*right*) chains from sorted cells (y-axis) of all clonotypes previously identified by the CET analysis from this donor (independently of tetramer, using the same analysis and cluster identities shown Figure 5B). For each cluster containing ≥1 clonotype detectable in the sorted cells, the numbers of detectable / total clonotypes are listed at the top of the plot. Highlighted in yellow are the 5 CETs identified in cells cultured with the '14,15,16' peptide pool containing the GILGFVFTL sequence.

DONOR	HLA-A	HLA-A	HLA-B	HLA-B	HLA-C	HLA-C	HLA-DRB	HLA-DRB	HLA-DQB	HLA-DQB	HLA-DPB	HLA-DPB
ND1001	03:01	68:01	45:01	51:01	02:02	6:02	4:01	7:01	2:02	3:01	3:01	10:01
ND1002	24:02	31:02	35:01		4:01		4:07	9:01	3:02	3:03	4:01	4:02
ND1004	01:01	02:206	08:01	15:01	3:04	7:01	1:01	7:01	2:02	5:01	3:01	14:01
ND1005	02:01	29:02	44:02	44:03	5:01	16:01	07:01	13:01	2:02	6:03	11:01	14:01
ND1007	02:01	02:01	08:01	40:01	03:04	07:01	03:01	13:02	02:01	06:04	03:01	04:01
DONOR #30115	02:05	23:17	8:01	15:18	3:04	7:02	11:02	15:03	3:01	6:02	105:01	105:01
DONOR A12	01:01	74:01	13:02	57:01	06:02		04:01	07:01	03:02	03:03	NA	NA

Supplemental Table 1. HLA genotype of donors used in the study. Highlighted cells indicate alleles known (from previous studies) to present peptides recognized by TCR motifs detected here. NA indicates genotypes not available.

Peptide	Length	Sequence	precipitate level
01 of 60	15	1-MSLLTEVETYVLSII-15	medium
02 of 60	16	5-TEVETYVLSIIPSGPL-20	not soluble at all
03 of 60	15	9-TYVLSIIPSGPLKAE-23	none
04 of 60	15	13-SIIPSGPLKAEIAQR-27	none
05 of 60	15	17-SGPLKAEIAQRLESV-31	low
06 of 60	15	21-KAEIAQRLESVFAGK-35	none
07 of 60	15	25-AQRLESVFAGKNTDL-39	none
08 of 60	15	29-ESVFAGKNTDLEALM-43	none
09 of 60	15	33-AGKNTDLEALMEWLK-47	none
10 of 60	15	37-TDLEALMEWLKTRPI-51	none
11 of 60	15	41-ALMEWLKTRPILSPL-55	none
12 of 60	15	45-WLKTRPILSPLTKGI-59	none
13 of 60	15	49-RPILSPLTKGILGFV-63	none
14 of 60	15	53-SPLTKGILGFVFTLT-67	none
15 of 60	15	57-KGILGFVFTLTVPSE-71	none
16 of 60	15	61-GFVFTLTVPSERGLQ-75	none
17 of 60	15	65-TLTVPSERGLQRRRF-79	none
18 of 60	15	69-PSERGLQRRRFVQNA-83	none
19 of 60	15	73-GLQRRRFVQNALNGN-87	none
20 of 60	15	77-RRFVQNALNGNGDPN-91	none
21 of 60	14	82-NALNGNGDPNNMDR-95	none
22 of 60	15	85-NGNGDPNNMDRAVKL-99	none
23 of 60	15	89-DPNNMDRAVKLYKKL-103	none
24 of 60	15	93-MDRAVKLYKKLKREI-107	none
25 of 60	15	97-VKLYKKLKREITFHG-111	none
26 of 60	15	101-KKLKREITFHGAKEV-115	none
27 of 60	15	105-REITFHGAKEVSLSY-119	none
28 of 60	15	109-FHGAKEVSLSYSTGA-123	none
29 of 60	15	113-KEVSLSYSTGALASC-127	low
30 of 60	15	117-LSYSTGALASCMGLI-131	medium
31 of 60	15	121-TGALASCMGLIYNRM-135	high
32 of 60	15	125-ASCMGLIYNRMGTVT-139	none
33 of 60	15	129-GLIYNRMGTVTTEAA-143	low
34 of 60	15	133-NRMGTVTTEAAFGLV-147	none
35 of 60	15	137-TVTTEAAFGLVCATC-151	medium
36 of 60	15	141-EAAFGLVCATCEQIA-155	high
37 of 60	15	145-GLVCATCEQIADSQH-159	none
38 of 60	15	149-ATCEQIADSQHRSHR-163	none
39 of 60	14	154-IADSQHRSHRQMAT-167	none
40 of 60	16	157-SQHRSHRQMATTTNPL-172	none
41 of 60	15	161-SHRQMATTTNPLIRH-175	none
42 of 60	15	165-MATTTNPLIRHENRM-179	none
43 of 60	15	169-TNPLIRHENRMVLAS-183	none
44 of 60	15	173-IRHENRMVLASTTAK-187	none
45 of 60	15	177-NRMVLASTTAKAMEQ-191	none
46 of 60	15	181-LASTTAKAMEQMAGS-195	none
47 of 60	15	185-TAKAMEQMAGSSEQA-199	high
48 of 60	15	189-MEQMAGSSEQAAEAM-203	not soluble at all
49 of 60	15	193-AGSSEQAAEAMEVAN-207	medium
50 of 60	15	197-EQAAEAMEVANQTRQ-211	none
51 of 60	15	201-EAMEVANQTRQMVHA-215	medium
52 of 60	15	205-VANQTRQMVHAMRTI-219	none
53 of 60	16	209-TRQMVHAMRTIGTHPS-224	none
54 of 60	15	213-VHAMRTIGTHPSSSA-227	none
55 of 60	15	217-RTIGTHPSSSAGLKD-231	none
56 of 60	15	221-THPSSSAGLKDDLLE-235	none
57 of 60	15	225-SSAGLKDDLLENLQA-239	high
58 of 60	15	229-LKDDLLENLQAYQKR-243	none
59 of 60	15	233-LLENLQAYQKRMGVQ-247	none
60 of 60	16	237-LQAYQKRMGVQMQRFK-252	none

Supplemental Table 2. Influenza Matrix 1 (FluM1) peptides sequences and solubility. Entries are colored according to peptide group. Yellow=cluster 1, gray=cluster 2, green=cluster 3.

<table>
<tr><th>Donor</th><th colspan="3">CD4+ T cell response</th><th colspan="3">CD8+ T cell response</th></tr>
<tr><td></td><td>C1</td><td>C2</td><td>C3</td><td>C1</td><td>C2</td><td>C3</td></tr>
<tr><td>ND1001</td><td style="background:#ccc"></td><td style="background:#ccc"></td><td style="background:#ccc"></td><td style="background:#ccc"></td><td></td><td></td></tr>
<tr><td>ND1002</td><td style="background:#ccc"></td><td style="background:#ccc"></td><td style="background:#ccc"></td><td></td><td></td><td></td></tr>
<tr><td>ND1004</td><td style="background:#ccc"></td><td style="background:#ccc"></td><td style="background:#ccc"></td><td></td><td></td><td></td></tr>
<tr><td>ND1005</td><td style="background:#ccc"></td><td style="background:#ccc"></td><td style="background:#ccc"></td><td style="background:#ccc"></td><td style="background:#ccc"></td><td></td></tr>
<tr><td>ND1007</td><td></td><td style="background:#ccc"></td><td style="background:#ccc"></td><td style="background:#ccc"></td><td></td><td></td></tr>
</table>

Supplemental Table 3. Screening of normal donors for Flu M1 peptide region-specific $CD4^+$ or $CD8^+$ T cell responses to anti-CD40 11B6-CD40L: M1 stimulation in PBMC cultures as described for ND1004 and ND1005 in Figure 1. The shaded boxes indicate that anti-CD40 11B6-CD40L: M1 stimulation (0.01-0.1 nM) elicited $IFN\gamma^+$ and $TNF\alpha^+$ T cells upon re-stimulation with the indicated peptide clusters (C1-3), while the same PBMCs cultured with equivalent or higher doses of M1 protein did not elicit $IFN\gamma^+$ and $TNF\alpha^+$ T cells.

analysis name	donor	culture antigen	sequence_name_R1	cell input	number of read pairs
JK1503posIL2only	30115	no antigen	HUMAN-JK1503posIL2-098_S1_L001_R1_001.fastq.gz	1.00E+06	1,009,368
JK1503poslysate	30115	Mtb lystae	HUMAN-JK1503posMtblysate-098_S2_L001_R1_001.fastq.gz	1.00E+06	1,435,268
NT_1_deep	ND1001	no culture (ex vivo)	HUMAN-Donor1NoTreatment-097_S5_L001_R1_001.fastq.gz	1.00E+06	4,684,816
NT_1	ND1001	no culture (ex vivo)	HUMAN-Donor1NoTreatmentA-097_S1_L001_R1_001.fastq.gz	1.00E+07	153,544
NT_2_deep	ND1002	no culture (ex vivo)	HUMAN-Donor2NoTreatment-097_S7_L001_R1_001.fastq.gz	1.00E+06	114,771
NT_2	ND1002	no culture (ex vivo)	HUMAN-Donor2NoTreatmentA-097_S4_L001_R1_001.fastq.gz	2.00E+06	2,908,348
P10P11P12.1	ND1004	peptides 10+11+12	HUMAN-23Pep1011121-097_S36_L001_R1_001.fastq.gz	2.00E+05	274,957
P10P11P12.2	ND1004	peptides 10+11+12	HUMAN-23Pep1011122-097_S39_L001_R1_001.fastq.gz	2.00E+05	407,871
P123.1	ND1004	peptides 1+2+3	HUMAN-23Pep1231-097_S43_L001_R1_001.fastq.gz	2.00E+05	246,620
P123.2	ND1004	peptides 1+2+3	HUMAN-23Pep1232-097_S51_L001_R1_001.fastq.gz	2.00E+05	376,771
P14P15P16.1	ND1004	peptides 14+15+16	HUMAN-23Pep1415161-097_S28_L001_R1_001.fastq.gz	2.00E+05	348,246
P14P15P16.2	ND1004	peptides 14+15+16	HUMAN-23Pep1415162-097_S32_L001_R1_001.fastq.gz	2.00E+05	429,885
P24.1	ND1004	peptide 24	HUMAN-23Pep241-097_S37_L001_R1_001.fastq.gz	2.00E+05	479,013
P24.2	ND1004	peptide 24	HUMAN-23Pep242-097_S41_L001_R1_001.fastq.gz	2.00E+05	407,680
P26P27.1	ND1004	peptides 26+27	HUMAN-23Pep26271-097_S30_L001_R1_001.fastq.gz	2.00E+05	476,688
P26P27.2	ND1004	peptides 26+27	HUMAN-23Pep26272-097_S45_L001_R1_001.fastq.gz	2.00E+05	461,019
P32P33.1	ND1004	peptides 32+33	HUMAN-23Pep32331-097_S34_L001_R1_001.fastq.gz	2.00E+05	284,323
P32P33.2	ND1004	peptides 32+33	HUMAN-23Pep32332-097_S47_L001_R1_001.fastq.gz	2.00E+05	520,708
P43.1	ND1004	peptide 43	HUMAN-23Pep431-097_S46_L001_R1_001.fastq.gz	2.00E+05	424,843
P43.2	ND1004	peptide 43	HUMAN-23Pep432-097_S54_L001_R1_001.fastq.gz	2.00E+05	331,515
P45.1	ND1004	peptide 45	HUMAN-23Pep451-097_S49_L001_R1_001.fastq.gz	2.00E+05	431,281
P45.2	ND1004	peptide 45	HUMAN-23Pep452-097_S53_L001_R1_001.fastq.gz	2.00E+05	366,514
P5.1	ND1004	peptide 5	HUMAN-23Pep51-097_S48_L001_R1_001.fastq.gz	2.00E+05	470,650
P5.2	ND1004	peptide 5	HUMAN-23Pep52-097_S52_L001_R1_001.fastq.gz	2.00E+05	351,102
P53.1	ND1004	peptide 53	HUMAN-23Pep531-097_S44_L001_R1_001.fastq.gz	2.00E+05	336,232
P53.2	ND1004	peptide 53	HUMAN-23Pep532-097_S50_L001_R1_001.fastq.gz	2.00E+05	229,900
P60.1	ND1004	peptide 60	HUMAN-23Pep601-097_S38_L001_R1_001.fastq.gz	2.00E+05	398,584
P60.2	ND1004	peptide 60	HUMAN-23Pep602-097_S42_L001_R1_001.fastq.gz	2.00E+05	469,571
CD40Lflu01_4	ND1004	targeted M1	HUMAN_donor411B6CD40LFluM101nM_097_S3_L001_R1_001.fastq.gz	1.00E+06	2,072,034
CD40Lflu1_4	ND1004	targeted M1	HUMAN_donor411B6CD40LFluM11nM_097_S2_L001_R1_001.fastq.gz	1.00E+06	1,431,763
flu01_4	ND1004	untargered M1	HUMAN_donor4ControlFluM101nM_097_S5_L001_R1_001.fastq.gz	1.00E+06	1,850,465
flu1_4	ND1004	untargered M1	HUMAN_donor4ControlFluM11nM_097_S4_L001_R1_001.fastq.gz	1.00E+06	2,498,245
NT_4	ND1004	no culture (ex vivo)	HUMAN_donor4Notreatment_097_S1_L001_R1_001.fastq.gz	2.00E+06	13,134
NT_4_deep	ND1004	no culture (ex vivo)	HUMAN-Donor4NoTreatment-097_S2_L001_R1_001.fastq.gz	1.00E+06	3,897,412
expandedonly_4	ND1004	no antigen	HUMAN_donor4notreatmentbutexpansioncultured_097_S6_L001_R1_001.fastq.gz	1.00E+06	1,988,081
P1112.1	ND1005	peptides 11+12	HUMAN-21Pep11121-097_S2_L001_R1_001.fastq.gz	2.00E+05	531,112
P1112.2	ND1005	peptides 11+12	HUMAN-21Pep11122-097_S4_L001_R1_001.fastq.gz	2.00E+05	282,281
P1112.3	ND1005	peptides 11+12	HUMAN-21Pep11123-097_S9_L001_R1_001.fastq.gz	2.00E+05	483,534
P141516.1	ND1005	peptide 14+15+16	HUMAN-21Pep1415161-097_S3_L001_R1_001.fastq.gz	2.00E+05	448,136
P141516.2	ND1005	peptide 14+15+16	HUMAN-21Pep1415162-097_S8_L001_R1_001.fastq.gz	2.00E+05	354,092
P2.1	ND1005	peptide 2	HUMAN-21Pep21-097_S1_L001_R1_001.fastq.gz	2.00E+05	394,835
P2.2	ND1005	peptide 2	HUMAN-21Pep22-097_S5_L001_R1_001.fastq.gz	2.00E+05	599,761
P2.3	ND1005	peptide 2	HUMAN-21Pep23-097_S7_L001_R1_001.fastq.gz	2.00E+05	340,274
P2324.1	ND1005	peptides 23+24	HUMAN-21Pep23241-097_S10_L001_R1_001.fastq.gz	2.00E+05	97,289
P2324.2	ND1005	peptides 23+24	HUMAN-21Pep23242-097_S14_L001_R1_001.fastq.gz	2.00E+05	431,875
P2627.1	ND1005	peptides 26+27	HUMAN-21Pep26271-097_S20_L001_R1_001.fastq.gz	2.00E+05	339,428
P2627.2	ND1005	peptides 26+27	HUMAN-21Pep26272-097_S22_L001_R1_001.fastq.gz	2.00E+05	621,521
P31.1	ND1005	peptide 31	HUMAN-21Pep311-097_S6_L001_R1_001.fastq.gz	2.00E+05	442,763
P31.2	ND1005	peptide 31	HUMAN-21Pep312-097_S17_L001_R1_001.fastq.gz	2.00E+05	529,438
P31.3	ND1005	peptide 31	HUMAN-21Pep313-097_S18_L001_R1_001.fastq.gz	2.00E+05	495,291
P59.2	ND1005	peptide 59	HUMAN-21Pep592-097_S21_L001_R1_001.fastq.gz	2.00E+05	389,932
P59.3	ND1005	peptide 59	HUMAN-21Pep593-097_S25_L001_R1_001.fastq.gz	2.00E+05	407,107
CD40Lflu01_5	ND1005	targeted M1	HUMAN_donor511B6CD40LFluM101nM_097_S9_L001_R1_001.fastq.gz	1.00E+06	1,283,486
CD40Lflu1_5	ND1005	targeted M1	HUMAN_donor511B6CD40LFluM11nM_097_S8_L001_R1_001.fastq.gz	1.00E+06	2,839,039
flu01_5	ND1005	untargered M1	HUMAN_donor5ControlFluM101nM_097_S11_L001_R1_001.fastq.gz	1.00E+06	2,069,945
flu1_5	ND1005	untargered M1	HUMAN_donor5ControlFluM11nM_097_S10_L001_R1_001.fastq.gz	1.00E+06	2,027,023
NT_5_deep	ND1005	no culture (ex vivo)	HUMAN-Donor5NoTreatment-097_S1_L001_R1_001.fastq.gz	1.00E+06	4,099,780
NT_5	ND1005	no culture (ex vivo)	HUMAN_donor5Notreatment_097_S7_L001_R1_001.fastq.gz	1.00E+06	697,106
expandedonly_5	ND1005	no antigen	HUMAN_donor5notreatmentbutexpansioncultured_097_S12_L001_R1_001.fastq.gz	1.00E+06	1,253,635
GILtetramerSorted_5	ND1005	targeted M1	ND1005_SORT_TET_01nM_CD4011B6_CD40Ldoc_cohFluM1_S8_L001_R1_001.fastq.gz	4.00E+04	673,059
D0_7	ND1007	targeted M1	ND1007_KINETICS_D0_CD4011B6_CD40Ldoc_cohFluM1_S12_L001_R1_001.fastq.gz	1.00E+06	805,507
D2_7	ND1007	targeted M1	ND1007_KINETICS_D2_CD4011B6_CD40Ldoc_cohFluM1_S13_L001_R1_001.fastq.gz	1.00E+06	648,065
D4_7	ND1007	targeted M1	ND1007_KINETICS_D4_CD4011B6_CD40Ldoc_cohFluM1_S14_L001_R1_001.fastq.gz	1.00E+06	593,103
D6_7	ND1007	targeted M1	ND1007_KINETICS_D6_CD4011B6_CD40Ldoc_cohFluM1_S15_L001_R1_001.fastq.gz	1.00E+06	537,956
D8_7	ND1007	targeted M1	ND1007_KINETICS_D8_CD4011B6_CD40Ldoc_cohFluM1_S16_L001_R1_001.fastq.gz	1.00E+06	589,719
NS_7	ND1007	no antigen	ND1007_NO_STIM_CONTROL_S10_L001_R1_001.fastq.gz	1.00E+06	1,061,943

Supplemental Table 4. Cell count inputs and final read depths for all samples analyzed.
